# Applying the WHO ICD-MM classification system to maternal deaths in a tertiary hospital in Nigeria: A retrospective analysis from 2014–2018

**DOI:** 10.1371/journal.pone.0244984

**Published:** 2021-01-04

**Authors:** Godwin O. Akaba, Obiageli E. Nnodu, Nessa Ryan, Emmanuel Peprah, Teddy E. Agida, Dilly O. C. Anumba, Bissallah A. Ekele

**Affiliations:** 1 Department of Obstetrics and Gynaecology, College of Health Sciences, University of Abuja/University of Abuja Teaching Hospital, Gwagwalada, Nigeria; 2 Department of Haematology and Blood Transfusion, College of Health Sciences, University of Abuja/University of Abuja Teaching Hospital, Gwagwalada, Nigeria; 3 New York University School of Global Public Health, New York University, New York, NY, United States of America; 4 Academic Unit of Reproductive and Developmental Medicine, University of Sheffield, Sheffield, United Kingdom; Liverpool School of Tropical Medicine, UNITED KINGDOM

## Abstract

**Background:**

Addressing the problem of maternal mortality in Nigeria requires proper identification of maternal deaths and their underlying causes in order to focus evidence-based interventions to decrease mortality and avert morbidity.

**Objectives:**

The objective of the study was to classify maternal deaths that occurred at a Nigerian teaching hospital using the WHO International Classification of Diseases Maternal mortality (ICD-MM) tool.

**Methods:**

This was a retrospective observational study of all maternal deaths that occurred in a tertiary Nigerian hospital from 1^st^ January 2014 to 31^st^ December,2018. The WHO ICD-MM classification system for maternal deaths was used to classify the type, group, and specific underlying cause of identified maternal deaths. Descriptive analysis was performed using Statistical Package for Social Sciences (SPSS). Categorical and continuous variables were summarized respectively as proportions and means (standard deviations).

**Results:**

The institutional maternal mortality ratio was 831/100,000 live births. Maternal deaths occurred mainly amongst women aged 25–34 years;30(57.7%), without formal education; 22(42.3%), married;47(90.4%), unbooked;24(46.2%) and have delivered at least twice;34(65.4%). The leading causes of maternal death were hypertensive disorders in pregnancy, childbirth, and the puerperium (36.5%), obstetric haemorrhage (30.8%), and pregnancy related infections (17.3%). Application of the WHO ICD-MM resulted in reclassification of underlying cause for 3.8% of maternal deaths. Postpartum renal failure (25.0%), postpartum coagulation defects (17.3%) and puerperal sepsis (15.4%) were the leading final causes of death. Among maternal deaths, type 1, 2, and 3 delays were seen in 30(66.7%), 22(48.9%), and 6(13.3%), respectively.

**Conclusion:**

Our institutional maternal mortality ratio remains high. Hypertensive disorders during pregnancy, childbirth, and the puerperium and obstetric haemorrhage are the leading causes of maternal deaths. Implementation of evidence-based interventions both at the hospital and community levels may help in tackling the identified underlying causes of maternal mortality in Nigeria.

## Introduction

Nigeria was ranked fourth amongst countries with the highest maternal mortality ratio (MMR) in 2017 after South Sudan, Chad and Sierra Leone (WHO, 2019) [1]. Despite recent efforts by the Federal government and other relevant stakeholders, Nigeria’s current MMR of 512/100,000 live births still remains one of the highest globally [2]. The International Classification of Diseases (ICD-10) defines maternal death as “the death of a woman while pregnant or within 42 days of the end of pregnancy, irrespective of the duration and site of the pregnancy, from any cause related to or aggravated by the pregnancy or its management, but not from accidental or incidental causes” [3]. Maternal death audit and reviews crucially facilitate the identification and analysis of the causes of and contributory factors to these deaths. Such audits contribute to improved quality of care, and may be key to attaining the Sustainable Development Goals (SDGs) [4, 5].

The application of the World Health Organization’s International Classification of Diseases—Maternal Mortality (WHO ICD-MM) classification system to maternal deaths is intended to facilitate the consistent collection, analysis and interpretation of information on maternal deaths. These are vital towards health system strengthening, intervention planning, and policy generation for prevention of maternal deaths [6]. The ICD-MM classification of causes of maternal death includes three levels of classification (type, group and underlying cause of death). The type of maternal death could be direct, indirect, or unspecified maternal death. Direct obstetric deaths are those resulting from obstetric complications of the pregnancy state (pregnancy, labour and puerperium), from interventions, omissions, incorrect treatment, or from a chain of events resulting from any of the above while indirect obstetric deaths are those resulting from previous existing disease or disease that developed during pregnancy and which was not due to direct obstetric causes, but which was aggravated by physiologic effects of pregnancy. Unspecified maternal deaths are deaths during pregnancy, childbirth and the puerperium where the underlying cause is unknown or was not determined [6]. The groups are as follows: (1: pregnancies with abortive outcome, 2: hypertensive disorders in pregnancy, childbirth, and the puerperium, 3: obstetric haemorrhage, 4: pregnancy-related infection, 5: other obstetric complications, 6: unanticipated complications of management, 7: non-obstetric complications, 8: unknown/undetermined, 9: coincidental causes) [6]. The underlying cause of death is defined as the disease or injury which initiated the cascade of morbid events leading directly to the death or the circumstances of the accident or violence which produced the fatal injury [6, 7].

Although facility-based reviews are conducted in most tertiary institutions in Nigeria, application of the ICD MM classifications during maternal death reviews is still not universally practiced. This makes it difficult for comparing data between these facilities for effective planning and targeted prevention of further maternal deaths. Therefore, the objective of this study was to apply the WHO ICD-MM classification system in analyzing the underlying causes of maternal deaths at a tertiary hospital in Nigeria. It is hoped that findings from this study will clarify and standardize reporting of the underlying causes of maternal deaths in this tertiary referral hospital towards targeted interventions. Additionally, it could set a benchmark for wider adoption and consistent use of the ICD-MM classification for reporting maternal deaths in Nigeria.

## Materials and methods

This was a retrospective review of maternal deaths that occurred at the University of Abuja Teaching Hospital from 2014–2018. Case folders and maternal death surveillance review forms were used for this study. The study was approved by the Research Ethics committee of the University of Abuja Teaching Hospital (Approval number: UATH/HREC/PR/2020/003/007). Informed consent was not necessary as the data were analysed anonymously.

### Study setting

The study was conducted at the Department of Obstetrics and Gynaecology of the University of Abuja Teaching Hospital (UATH), Gwagwalada, Abuja, a 400-bed tertiary hospital in the nation’s capital. The Federal Capital Territory (F.C. T) covers an area of 8,000 square kilometers and receives referrals from other hospitals in the F.C.T as well as from four neighboring states. The department undertakes about 1500–2000 deliveries annually and conducts monthly review/audit of all maternal deaths in order to understand the causes and events surrounding them. The rationale for this quality control effort is to avert maternal deaths in the facility by addressing identified factors and improving quality of care in the facility as well as the referring facilities.

### Study design

A retrospective observational study of all maternal deaths that occurred in the hospital from 1^st^ January 2014 to 31^st^ December 2018 was carried out.

### Data collection/analysis

We reviewed the case records and completed Maternal Death Surveillance and Response (MDSR) forms of all maternal deaths at the University of Abuja Teaching Hospital. A pretested data collection tool adapted from the MDSR questionnaire was used to extract the following Information: age, educational status (No formal education/primary/secondary/tertiary), marital status(married/single/divorced), parity (nullipara/para 1/para 2/para 3/para 4/para 5 and above), booking status/place of booking (unbooked/booked at teaching hospital, secondary health center, primary health center or private hospital), type of referring facility, duration of hospital stay, presence of delays and types of delays (type 1, 2 or 3), underlying cause of death and final cause of death. Based on delay experienced in health seeking (type 1), transit to the facility, and delay at the facility, type of delay was retrieved from the patient note indicated in the medical chart, which is also discussed and categorized during periodic maternal mortality review meetings at the facility. Maternal deaths were categorized as primary delay if the women presented to the hospital later than 24 hours from recognition of a pregnancy complication while women who took more than 4 hours from when they left their homes or referral hospital to reach UATH were considered to have had secondary delay. The tertiary delay was present if the pregnant woman did not receive care or was not admitted within 30 minutes of presentation at the teaching hospital. For this study, a woman was considered to have booked for pregnancy care if she registered and attended ANC at any health facility. Such health facility was designated as the place of booking for that particular patient. The causes of deaths documented in the folder as well as on the MDSR booklet were reviewed for correctness and consistency by GOA and two senior residents in Obstetrics and Gynaecology. Thereafter the WHO ICD-MM classification system for maternal deaths was used to classify the identified causes of death into types, groups, underlying causes, and corresponding codes. A total of 64 maternal deaths occurred during the period under review out of which only 52 case folders could be retrieved for comparison with information on the MDSR forms for this study. The total number of deliveries as well as live births during the period under review was extracted from the labour ward registers and used to calculate the maternal mortality ratio.

Data was entered into excel and descriptive analysis was performed using Statistical Package for Social Sciences (SPSS) (IBM, NY, version 20). Only the 52 cases of maternal deaths whose folders were retrievable and compared with information on the MDSR forms were used for analysis except for the calculation of the institutional MMR in which the total number of maternal deaths of 64 was used.

All maternal deaths were classified using the ICD-MM system (type, group and underlying cause), types of delay associated with each maternal death. The difference in underlying cause of maternal deaths in the case notes, MDSR forms and the underlying cause of death using the ICD-MM system was described. Categorical and continuous variables were summarized respectively as proportions and means (standard deviations).

## Results

### Sample characteristics

During the five years period under review, a total of 64 maternal deaths occurred amongst 7,909 births and 7,703 live births. This gave a MMR of 831/100,000 live births. Maternal deaths occurred mainly amongst women aged 25–34 years; 30(57.7%), without formal education; 22(42.3%), married; 47(90.4%), unbooked; 24(46.2%) and have delivered at least twice; 34(65.4%) (Table 1). The public secondary health facilities were the main source of referrals to the Teaching Hospital comprising 17(32.7%) cases, followed by the private clinics which accounted for 16(30.8%). Eight (15.4%) patients arrived directly from their homes, 6(11.5%) from primary health centers (PHC) and 5(9.6%) were referred from another tertiary hospital. The majority, 25(48.1%), presented to our unit either before 28 weeks' gestation due to pregnancy complications or just before onset of labour, 19(36.5%) presented in the postpartum period and the remaining 8(15.4%) presented in the intrapartum period. The mean duration of hospital stay was 4.3 ± 6 days and 25(48.1%) women died within the first 24 hours of presentation.

**Table 1:** Socio-demographic characteristics of cases of maternal deaths at University of Abuja Teaching Hospital, Abuja, Nigeria, 2014–2018 (n = 52).

Variables	Frequency	%
Age (years) mean(±SD)	28.9±5.8	
Age group (years)		
≤ 19	2	3.8
20–24	8	15.4
25–29	13	25.0
30–34	17	32.7
≥ 35	12	23.1
Educational status		
No formal education	22	42.3
Primary	10	19.2
Secondary	10	19.2
Graduate	10	19.2
Marital status		
Married	47	90.4
Single	4	7.7
Divorced	1	1.9
Parity		
Nullipara	9	17.3
Para1	9	17.3
Para2	15	28.8
Para3	12	23.1
Para4	7	13.5
Hospital stay (days)		
≤24 hrs	25	48.1
2–7	21	40.4
8–13	2	3.8
14–19	2	3.8
≥20	2	3.8
Booking status		
Unbooked	24	46.2
At teaching hospital	3	5.8
At primary health centre	5	9.6
At secondary health centre	7	13.5
At private facility	13	25.0
Delivery mode		
Undelivered	4	7.7
Vaginal delivery	23	44.2
C-section	25	48.1
Combinations of type of delay		
No identified delay	20	38.5
Type 1 delay alone	7	13.5
Type 2 delay alone	-	-
Type 3 delay alone	1	1.9
Types 1&2 delays	19	36.5
Types 1&3 delays	2	3.8
Types 2&3 delays	1	1.9
Types 1,2&3 delays	2	3.8

### Type of delay

There was delay in seeking care in 30 (66.7%), delay in reaching the referred hospital due to either transportation or late referrals in 22 (48.9%), while delay in diagnosis or instituting treatment occurred in 6 (13.3%). The various combinations of the type of delay seen amongst the cases of maternal deaths are also shown in Table1.

### Cause of maternal death

Overall, 49/52(94.2%) were direct maternal deaths while the remaining 3/52(6.0%) were indirect.

Before application of the WHO ICD-MM classification system, the major underlying cause of death was preeclampsia/eclampsia occurring in 19(36.5%) of cases. Group 2 (i.e., hypertensive disorders in pregnancy, childbirth, and the puerperium) remained the major underlying cause of maternal deaths after reclassification with WHO ICD-MM without change in proportion. The disaggregation showed that severe preeclampsia alone contributed 10/52(19.2%), eclampsia in pregnancy, 8/52(15.4%) and eclampsia in the puerperium, 1/52(1.9%) to the total burden of maternal deaths and 10/19(52.6%), 8/19(42.1%) and 1/19(5.3%) respectively to deaths from hypertensive disorders of pregnancy.

Obstetric haemorrhage was the second major cause of maternal deaths in 15(28.8%) of cases. The contribution of group 3 (i.e., obstetric haemorrhage) increased from 28.8% to 30.8% due to reclassification of one case of ruptured uterus which was initially classified as puerperal sepsis because she finally died of sepsis. Application of the WHO ICD-MM classification system also showed that obstetric haemorrhage from uterine atony,8/52(15.4%) and ruptured uterus,5/52(9.6%) were the main underlying causes of maternal deaths from group 3, accounting respectively for 8/16(50.0%) and 5/16(31.3%) of deaths from obstetric haemorrhage. A case of obstructed labour which is a contributory cause of death was initially categorized as an underlying cause of death but was reclassified using the ICD-MM under group 6 (i.e., unanticipated complications of management: O74.2 cardiac complications of anaesthesia during labour and delivery). The underlying causes of death before and after the application of the WHO ICD-MM classification system are shown in Table 2 while the subgroup classification of underlying causes of maternal deaths with ICD-MM codes are shown in Table 3.

**Table 2:** Underlying causes of death before and after application of WHO ICD-MM classification among cases of maternal death at University of Abuja Teaching Hospital, Abuja, Nigeria, 2014–2018 (n = 52).

Underlying cause of death (N = 52)	Before application of ICD-MM Frequency (%)	After application of ICD-MM Frequency (%)
Preeclampsia/eclampsia	19(36.5)	19(36.5)
Obstetric haemorrhage	15(28.8)	16(30.8)
Puerperal sepsis	10(19.2)	9(17.3)
Unsafe abortion	4(7.7)	4(7.7)
Anaemic heart failure	2(3.8)	2(3.8)
Obstructed labour	1(1.9)	-
Severe malaria	1(1.9)	1(1.9)
Cardiac complication of anaesthesia	-	1(1.9)

**Table 3:** Subgroup classification of underlying causes of maternal deaths with WHO ICD-MM codes among cases of maternal death at University of Abuja Teaching Hospital, Abuja, Nigeria, 2014–2018 (n = 52).

Type of maternal death	Group number/name	Code	Underlying cause of death	N (%)
Direct	1.Pregnancies with abortive outcomes	O07.5	Other and unspecified failed attempted abortion complicated by genital tract and pelvic infection	4(7.7)
	2.Hypertensive disorders of pregnancy, childbirth and puerperium	O14.1	Severe pre-eclampsia	10(19.2)
		O15.0	Eclampsia in pregnancy	8 (15.4)
		O15.2	Eclampsia in the puerperium	1(1.9)
	3.Obstetric haemorrhage	O72.1	Other immediate postpartum haemorrhage (atonic uterus)	8(15.4)
		O71.1	Rupture of uterus during labour	5(9.6)
		O44.1	Placenta praevia with haemorrhage	1(1.9)
		O67.8	Other intrapartum haemorrhage (Excessive intrapartum haemorrhage)	1(1.9)
		O71.3	Obstetric laceration of cervix	1(1.9)
	4.Pregnancy related infections	O85	Puerperal sepsis	7(13.5)
		O86.0	Infection of obstetric surgical wound	2(3.8)
	5.Other obstetric complications	Nil		
	6.Unanticipated complications of management	O74.2	Cardiac complication of anaesthesia during labour and delivery	1(1.9)
Indirect	7.Non-obstetric complications	O99.0	Anaemia complicating pregnancy, childbirth and the puerperium	2(3.8)
		O98.6	Protozoal diseases complicating pregnancy, childbirth and the puerperium	1(1.9)
Maternal death: unspecified	8.Unknown/undetermined	Nil		
Death during pregnancy, childbirth and the puerperium	9. Coincidental causes	Nil		

## Discussion

Our study shows that the MMR remains high within a tertiary referral hospital in Nigeria’s Federal Capital Territory, although it has reduced from the 2123 maternal deaths/100,000 live births reported in the same facility about a decade ago [8]. The high MMR reported is not unexpected, as it is a tertiary referral hospital with majority of the women being referred with severe complications requiring more specialized care. Although higher than the current national MMR of 512 maternal deaths /100,000 live births [2], it is much lower than 1640/100,000 live births recorded in a similar hospital-based retrospective analysis of maternal deaths at Ife, South Western Nigeria [9] and 2,085/100,000 live births(range: 877–4,210 per 100,000 births) reported in a multicenter review of maternal deaths in eight secondary level hospitals across eight states in four geopolitical zones in Nigeria [10]. The high burden of maternal deaths in this study and in other tertiary health institutions in Nigeria underscores the need for sustained efforts at addressing the problem of maternal mortality towards realization of SDG3 target 3.1 (i.e., to reduce the global maternal mortality ratio to less than 70/100,000 live births).

Majority of these women died in their prime of life, are unbooked, and are educationally disadvantaged. This conforms with previous reports from Nigeria [9, 10–13]. Lack of maternal education has been reported to increase the risk of maternal deaths [14–16]. Uneducated women may find it difficult to recognize danger signs, are less economically empowered to seek care, have poor health seeking behaviours, and are highly dependent on the approval of their husband or male head of household to access health care. They therefore do not attend antenatal care and present late to maternal healthcare facilities when complications have already supervened [17].

It is instructive to note that only few women were referred from the primary health centers in this study. It is likely that Nigerian governments interventions at the primary health care levels are yielding fruits leading to early referrals from these levels of care. On the other hand, it may be that women are not patronizing the primary health centers due to poor quality of care, which may lead to high patronage of the secondary health care and private hospitals with increasing referrals from them. Despite the huge contribution of the private sector to health care service delivery in Nigeria, this sector has not received much attention from the federal and state governments in the area of capacity building and trainings of health care workers towards improving maternal and newborn health. The same goes for the primary and secondary health institutions which are mainly supported by the state governments as “health” falls under the concurrent legislative list which gives the state government the powers to enact laws and systems that will guide operation of its health system. In principle, state governments are responsible for secondary hospital care. There must be a renewed focus on training of health care workers in the private sector, primary, and secondary health facilities on prevention and management of obstetrics emergencies towards reducing the burden of maternal mortality in Nigeria.

Hypertensive disorders of pregnancy, childbirth and puerperium was the leading cause of maternal deaths in this study, while obstetric haemorrhage was the second most common cause of maternal deaths. A previous report on causes of maternal death in the same institution in 2011 identified puerperal sepsis and obstetric haemorrhage as the leading and second most common cause of maternal deaths, respectively [8]. The decline in the contribution of puerperal sepsis may be due to the availability and use of highly effective antibiotics for women at risk of or diagnosed to have puerperal sepsis. On the other hand, there was an increase in the contribution of obstetric haemorrhage from 15.3% in 2011 to 28.8% in the current study and even higher to 30.8% using the ICD 10-MM classification.

Although the leading causes of maternal deaths may vary from one region to the other, hypertensive disorders of pregnancy, childbirth and puerperium or obstetrics haemorrhage have persistently been amongst the top two causes of maternal deaths in Nigeria [11, 18–21]. The use of the WHO ICD-MM classification of maternal deaths clearly demonstrated the contributions of severe preeclampsia and eclampsia in pregnancy as the major disease conditions under hypertensive disorders of pregnancy, childbirth and puerperium that need to be addressed. This is also true for other immediate postpartum haemorrhage (i.e., atonic uterus) and ruptured uterus as conditions that need to be targeted for deployment of evidence-based interventions towards averting maternal deaths from obstetric haemorrhage.

Application of the WHO 1CD-MM classification enabled proper reclassification of 3.8% of causes of maternal deaths. This was not remarkable and comparable to previous findings in which using the new ICD-MM classification system for attribution of causes of maternal deaths showed reclassification in 3.1% to 4.6% of cases depending on the groups or data set reviewed from five countries [7]. Another study from Malawi [22] when compared with our study however showed a high rate of variation between what was recorded by health workers using the MDR forms and findings using ICD-MM classification. This can be attributed to the challenges of improper and incomplete filling of MDR forms by health workers, non-regular training of health workers responsible for completing MDR forms and occasional delays in filling MDR forms in the latter study. These could make determination and documentation of underlying cause of death challenging. While the reclassification increased the events attributable to obstetric haemorrhage on the one hand, it also enabled identification of cardiac complications of anaesthesia during labour and delivery, a hitherto rarely reported underlying cause of maternal death. As an immediate cause of death, complication of anaesthesia contributed 7.7% to maternal deaths and is much higher than 2.8% reported in a systematic review and metanalysis of anaesthesia related maternal mortality in low-income and middle-income countries [23]. The cases in this study had spinal anaesthesia but a previous study [24] had identified reasons for anaesthesia related maternal mortality, including the role of general anaesthesia, inadequate supervision of trainee anaesthetists, and a lack of appropriate monitors. The contribution of anaesthesia related deaths to increasing maternal mortality in Nigeria and other LMIC calls for an in-depth review and mitigation of established risk factors and adherence to safety recommendations like the routine use of the World Health Organization (WHO) surgical check list. A study from Nigeria had reported that only 62.7% of surveyed anaesthetists routinely use this important safety tool [25].

The strength of our study lies in the multidisciplinary review of maternal deaths and the rigor that is associated with the process. The study is however limited by the sample size of 52 which is somewhat small to allow any additional analyses than what is presented here, but further studies aggregating maternal healthcare data across the Federal Capital Territory are needed to examine the various factors contributing to maternal death in this setting. We were unable to retrieve the case folders of all the maternal deaths that occurred during the period.

## Conclusions

In conclusion, the MMR at the only teaching Hospital in Nigeria’s Federal Capital Territory is still high with hypertensive disorders of pregnancy and obstetric haemorrhage being the leading causes of maternal deaths. Application of the WHO ICD 10-MM classification resulted in the reclassification of 3.8% of underlying causes of death and more importantly helped in identifying the specific disease entities in each of the groups that needs to be targeted towards reducing maternal deaths in Nigeria. The problems of severe preeclampsia, eclampsia in pregnancy and obstetric haemorrhage from uterine atony needs to be addressed if Nigeria must meet the SDG3.In addressing the above problems, contextual factors like patient education, prompt access to health care facilities, affordability of services, availability of medicines, skills of the health workers and quality of care at the health facilities should be well considered. We also recommend that studies evaluating the causes of maternal mortality, should apply the WHO ICD-10:MM classification for uniformity of reporting and clarity of underlying and final causes of deaths towards deploying necessary interventions to prevent maternal deaths.

## Supporting information

S1 Data (XLS)
